# Tagging SNPs in the *MTHFR* Gene and Risk of Ischemic Stroke in a Chinese Population

**DOI:** 10.3390/ijms15058931

**Published:** 2014-05-20

**Authors:** Bao-Sheng Zhou, Guo-Yun Bu, Mu Li, Bin-Ge Chang, Yi-Pin Zhou

**Affiliations:** 1 Department of Neurosurgery, Tianjin First Center Hospital, Tianjin 300192, China; E-Mails: zhoubaosheng99@sohu.com (B.-S.Z.); limu@team301.org (M.L.); changbinge@team301.org (B.-G.C.); 2 Department of Spine Surgery, Tianjin Hospital, Tianjin 300211, China; E-Mail: bgy860@sohu.com; 3 Department of Neurosurgery, Tianjin Third Central Hospital, Tianjin 300170, China

**Keywords:** ischemic stroke, *MTHFR*, polymorphism, homocysteine

## Abstract

Stroke is currently the leading cause of functional impairments worldwide. Folate supplementation is inversely associated with risk of ischemic stroke. Methylenetetrahydrofolate reductase (MTHFR) is an important enzyme involved in folate metabolism. The aim of this study is to examine whether genetic variants in *MTHFR* gene are associated with the risk of ischemic stroke and fasting total serum homocysteine (tHcy) level. We genotyped nine tag SNPs in the *MTHFR* gene in a case-control study, including 543 ischemic stroke cases and 655 healthy controls in China. We found that subjects with the rs1801133 TT genotype and rs1801131 CC genotype had significant increased risks of ischemic stroke (adjusted odds ratio (OR) = 1.82, 95% confidence interval (CI): 1.27–2.61, *p* = 0.004; adjusted OR = 1.99, 95% CI: 1.12–3.56, *p* = 0.01) compared with subjects with the major alleles. Haplotype analysis also found that carriers of the *MTHFR* CTTCGA haplotype (rs12121543-rs13306553-rs9651118-rs1801133-rs2274976-rs1801131) had a significant reduced risk of ischemic stroke (adjusted OR = 0.53, 95% CI: 0.35–0.82) compared with those with the CTTTGA haplotype. Besides, the *MTHFR* rs1801133 and rs9651118 were significantly associated with serum levels of tHcy in healthy controls (*p* < 0.0001 and *p* = 0.02). These findings suggest that variants in the *MTHFR* gene may influence the risk of ischemic stroke and serum tHcy.

## 1. Introduction

Stroke is a devastating neurological disease that mainly caused by abnormal perfusion of brain tissue, which ranked the second leading cause of death in people older than 60 years worldwide [1]. Over the last 4 decades (1970–2008), stroke incidence declined 42% in the developed countries, while the incidence rates in developing countries have increased by >100% and exceed that of the developed countries by 20% [2]. According to recent statistics, the annual stroke mortality rate in China is approximately 157 per 100,000, which has become the leading cause of death and adult disability [3]. Ischemic stroke is the most common type of stroke in China. Data from 1996 to 2000 showed that ischemic stroke accounts for about two thirds of strokes in China [4].

The major risk factors for stroke in China include hypertension, dyslipidemia, obesity, diabetes and smoking [5]. Data have also shown that certain nutrients and vascular endothelial regulation factors, such as folic acid and Kruppel-like factor 2, are protective factor for ischemic stroke [6]. Folic acid intake appears to reduce the risk of stroke through the regulation of plasma homocysteine concentration, which was a strong and independent risk factor for stroke [7]. It was reported that a 25% lower usual homocysteine level was associated with a 19% lower risk of stroke [8]. Several polymorphisms in homocysteine regulatory genes, such as *MTRR*, *SHMT1* and *TCN2*, have also been found to have impacts on plasma homocysteine level and ischemic stroke risk [9]. MTHFR (methylenetetrahydrofolate reductase), a folate-dependent enzyme, plays an important role in the conversion of the amino acid homocysteine to another amino acid, methionine, by converting 5,10-methylenetetrahydrofolate to 5-methyltetrahydrofolate. Studies have found that specific genetic mutations in the *MTHFR* gene could lead to change of MTHFR enzyme activity [10,11]. Previous epidemiologic studies have shown that genetic mutations in *MTHFR* gene may be related to cancers, type 2 diabetes, cardiovascular diseases, hypertension, *etc*. [12–14]. However, data on MTHFR genotypes and susceptibility to ischemic stroke in Chinese population are relatively rare, lack consistency and mainly focused on the most studied SNPs (C677T and A1298C) [15–19].

To help clarify whether the *MTHFR* variants are associated with susceptibility of ischemic stroke and total serum homocysteine (tHcy) level, we examined nine tag SNPs (single nucleotide polymorphism) in the *MTHFR* gene (rs12121543, rs13306561, rs13306553, rs9651118, rs1801133, rs2274976, rs4846048, rs1801131, rs17037396) in a case-control study in the Chinese population.

## 2. Results

Characteristics of the study subjects are shown in Table 1. Cases and controls were evenly matched by age and gender. Subjects were more likely to smoke cigarettes (41.3% *vs*. 32.8%), have diabetes (25.2% *vs*. 12.4%) and hypertension (72.4% *vs*. 33.9%). Besides, the subjects have significantly lower levels of serum HDL-C and heart rate, and higher levels of serum LDL-C than that of the controls.

The associations of *MTHFR* variants and risk of ischemic stroke are presented in Table 2. The genotype distributions of these nine polymorphisms showed no deviation from the expected Hardy–Weinberg equilibrium among controls (*p* > 0.05). Of these SNPs, TT genotype of rs1801133 and CC genotype of rs1801131 conferred significant higher risk of ischemic stroke (OR = 1.64, 95% CI: 1.16–2.31, *p* = 0.004; OR = 1.97, 95% CI: 1.13–3.45, *p* = 0.01) compared with CC and AA genotypes. These associations were still significant after adjustment for other risk factors (age, smoking, hypertension and diabetes). None of the other SNPs examined was associated with the risk of ischemic stroke.

Six SNPs in the *MTHFR* gene (rs12121543, rs13306553, rs9651118, rs1801133, rs2274976, rs1801131) were in linkage disequilibrium with D′ ranging from 0.61 to 0.99 and *r*^2^ ranging from 0.03 to 0.84. Subjects carrying the *MTHFR* CTTCGA haplotype had a significant reduced risk of ischemic stroke (OR = 0.55, 95% CI: 0.36–0.84) compared with those carrying the CTTTGA haplotype. This association was still significant after adjustment for other risk factors (Table 3).

Finally, we investigated the associations between the *MTHFR* SNPs and serum tHcy levels in the control population. Carriers of the mutant alleles of rs1801133 were significantly associated with increased serum level of tHcy (C/T: 12.8 (10.9–14.6) mmol/L; T/T: 13.1 (10.0–16.8) mmol/L) compared with carriers of the CC genotype (12.5 (9.8–14.9) mmol/L). Rs9651118 T/C and C/C genotypes (12.0 (10.0–13.8) mmol/L; 11.5 (9.5–14.1) mmol/L) conferred significant decreased serum level of tHcy in controls compared with TT genotype (10.9 (9.2–12.8) mmol/L). These associations were also significant in multivariate ANCOVA analyses. None of the other studied SNPs were associated with serum tHcy level (Table 4).

## 3. Discussion

In this molecular epidemiologic study, polymorphisms in the *MTHFR* gene were fully studied for their association with susceptibility to ischemic stroke and serum tHcy levels. We demonstrated that two genetic mutations (rs1801133 and rs1801131) in *MTHFR* gene were significantly associated with risk of ischemic stroke, and rs1801133 and rs9651118 were significantly associated with serum tHcy levels in our study population.

The *MTHFR* gene is located on chromosome 1 location p36.3 in humans. To date, over 40 point mutations in *MTHFR* gene have been identified, of which C677T (rs1801133) and A1298C (rs1801131) seem to have the most clinical significance. The *MTHFR* rs1801133 polymorphism involving C to T substitution at position 677 (C677T) that results in the conversion of alanine to valine. This missense mutation results in approximately 70% and 35% reduction of normal MTHFR enzyme activity in TT and CT genotype carriers, respectively [10]. MTHFR activity has an adverse effect on tHcy level. It was also reported that 677TT allele was associated with elevated tHcy levels, predominantly in individuals who have a low plasma folate level [20]. Higher homocysteine concentration has a negative effect on stroke [8]. Thus, our findings are biologically possible.

Although many studies focused on the association of *MTHFR* C677T mutation and risk of stroke, the results varied, which may partially due to different population groups and sample size. A meta-analysis showed that TT genotype of C677T had a 1.84-fold significantly increased risk of hemorrhagic stroke compared to CC genotype, and subgroup analyses by ethnicity further proved that this association existed both in Asian and Caucasian populations [21]. However, another meta-analysis including 152,797 individuals failed to found any relation between C677T and risk of ischemic stroke (OR = 1.23 95% CI: 0.61–1.47) [22]. Besides, data from a meta-analysis did not show any association between the *MTHFR* C677T molecular variant and risk of carotid dissection, which is recognized as a cause of stroke [23]. In our study, we found a positive correlation between C677T mutation and ischemic stroke risk, and the risk alleles were associated with significant higher level of tHcy in healthy controls.

A1298C (rs1801131) is a common mutation in the *MTHFR* gene that results in the conversion of adenine to cytosine, and this mutation also leads to a reduction in MTHFR enzyme activity. Several studies have been performed to evaluate the effects of *MTHFR* A1298C mutation on the risk of ischemic stroke, but obtained conflicting results [24–27]. The association between A1298C and serum homocysteine level was also controversial. Kumar *et al*. found that CC genotype conferred significant increased level of homocysteine (16.3 μmol/L) compared with AA genotype (14.4 μmol/L) in an Indian population, while this association was not found in a Scottish population [28,29]. Although we found significant association between A1298C and risk of ischemic stroke, we failed to find its relation with homocysteine level. The reason for these results still needs to be clarified.

Rs9651118 is located in the intron region of *MTHFR* gene, and the genetic function of this polymorphism is still unclear. It was reported that mutant allele of rs9651118 was associated with reduced lung cancer risk in never smokers, and a nominally significant association with schizophrenia in the form of haplotypes (rs1801133, rs17421511, rs17037396, and rs9651118) in the *MTHFR* gene in a Japanese population [30,31]. Moreover, the rs9651118 TT genotype conferred a significantly elevated level of tHcy compared with CC genotype in a population-based CoLaus study [32]. In our research, rs9651118 was not associated with risk of ischemic stroke, but we found carriers of TT genotype had significant higher level of tHcy relative to CC genotype carriers, which was in accordance with the previous study.

## 4. Material and Methods

### 4.1. Study Participants

In this study, we consecutively enrolled 543 ischemic stroke patients in Tianjin first center hospital, aged from 45 to 89 years, between June 2011 and June 2013. Clinical diagnoses of ischemic stroke were made by CT or MRI scans of the brain. Six hundred and fifty five healthy control subjects were selected during the same period and from the same hospital, and were frequency matched to the cases by age (5-year age groups) and gender. All controls were individuals free of ischemic stroke that determined by medical history, clinical examinations, or ultrasound screening. At enrollment, demographic characteristics, anthropometric measures, medical histories were collected from each subject by a trained interviewer using a structured questionnaire. Blood samples were collected after a 12-h overnight fast and then separated into serum, red blood cells, and buffy coat. Written informed consent was obtained from all enrolled participants and this study was approved by the Ethics Committee of Tianjin first center hospital.

### 4.2. SNP Selection

Tag SNPs were selected by searching Han Chinese data from the HapMap project using the Tagger program. The following criteria were used to identify tag SNPs: (a) SNPs located in the gene or within the 2-kb region flanking the gene; (b) a minor allele frequency ≥0.1; (c) other unselected SNPs could be captured by one of the tagging SNPs with a linkage disequilibrium of *r*^2^ ≥ 0.90; and (d) SNPs significantly associated with stroke in previous studies were preferred. As a result, a total of nine tag SNPs were identified.

### 4.3. Laboratory Tests

Serum total cholesterol (TC), low density lipoprotein-cholesterol (LDL-C), high density lipoprotein-cholesterol (HDL-C) and fasting total homocysteine (tHcy) levels were determined by commercial kits from the Nanjing Jiancheng Bio-company (Nanjing, China).

Genomic DNA was extracted from buffy coat using DNA Extraction Kit (Qiagen, Hilden, Germany). Genotyping was performed using the TaqMan assay on the ABI PRISM 7900HT Sequence Detection System (Applied Biosystems, Foster City, CA, USA), with dual fluorescent reporter probes VIC and FAM. The genotyping call rate was >95%, and the completion rate was >99%. The quality and potential misclassification of the genotyping were assessed by re-genotyping 5% of duplicate DNA samples that were randomly selected from the whole population and placed within the same reaction plates used for the study subjects. The concordance rate for the quality control samples was 100%.

### 4.4. Statistical Analysis

We used SAS software (version 9.3; SAS Institute, Inc., Cary, NC, USA) for the statistical analyses. χ^2^ statistics and the *t* test were used to evaluate case-control differences in the distribution of risk factors. Variables were tested for normality with Shapiro–Wilk statistics. Skewed data, including age, BMI, TC, LDL-C, HDL-C, tHcy and heart rate were log transformed and expressed as medians and interquartile ranges. The odds ratios (ORs) and 95% confidence intervals (CIs) for the associations between the SNPs and disease risk were estimated by unconditional logistic regression. Hardy–Weinberg equilibrium for genotypic distribution and linkage disequilibrium between loci were assessed by HaploView version 4.0 (Daly Lab at the Broad Institute, Cambridge, MA, USA) [33]. Associations between haplotypes (>1% frequency) and the risk of ischemic stroke were evaluated by computing OR and 95% CI using HAPSTAT, assuming an additive model, using the most common haplotype as the referent category [34]. Both univariate ANOVA and multivariate ANCOVA analyses adjusting for age, smoking, diabetes and hypertension were performed to determine the effects of the MTHFR polymorphisms on serum tHcy levels in healthy controls. A two tailed *p*-value of 0.05 was considered statistically significant.

## 5. Conclusions

In conclusion, the present study suggests *MTHFR* rs1801133 and rs1801131 were associated with ischemic stroke risk and *MTHFR* rs1801133 and rs9651118 may affect serum tHcy levels.

## Figures and Tables

**Table 1.** Selected characteristics of cases and controls.

Characteristics	Cases (*n* = 543)	Controls (*n* = 655)	*p* value
Age (year)	66 (61–70)	66 (60–70)	0.76
Sex (Male/Female)	346/197	402/253	0.42
Smoking (Yes/No)	224/319	215/440	0.003
Diabetes (Yes/No)	137/406	81/574	<0.001
Hypertension (Yes/No)	293/250	222/433	<0.001
BMI (kg/m^2^)	24.3 (22.9–25.8)	24.3 (23.0–25.7)	0.55
Total cholesterol (mmol/L)	4.70 (4.09–5.48)	4.14 (3.60–5.04)	0.82
HDL-C (mmol/L)	1.20 (1.09–1.51)	1.24 (1.08–1.56)	0.008
LDL-C (mmol/L)	2.82 (2.45–3.29)	2.48 (2.16–3.03)	<0.001
Heart rate (bmp)	72 (67–76)	74 (68–78)	<0.001

**Table 2.** Odds ratios (ORs) and 95% confidence intervals (CIs) for ischemic stroke in relation to polymorphisms of the methylenetetrahydrofolate reductase (*MTHFR*) gene.

SNP	Genotypes	Cases, *n* (%)	Controls, *n* (%)	OR (95% CI) †	OR (95% CI) ‡	P trend
rs12121543	CC	362 (66.8)	449 (68.7)	1.00	1.00	
	CA	159 (29.3)	185 (28.3)	1.04 (0.80–1.36)	0.99 (0.75–1.30)	
	AA	21 (3.9)	20 (3.1)	1.42 (0.75–2.71)	1.45 (0.74–2.84)	0.61
	CA + AA	180 (33.2)	205 (31.4)	1.08 (0.84–1.39)	1.03 (0.79–1.34)	
rs13306561	TT	432 (79.7)	529 (80.9)	1.00	1.00	
	TC	106 (19.6)	121 (18.5)	1.03 (0.76–1.39)	0.95 (0.69–1.29)	
	CC	4 (0.7)	4 (0.6)	1.27 (0.30–5.37)	1.36 (0.30–6.18)	0.67
	TC + CC	110 (20.3)	125 (19.1)	1.03 (0.77–1.39)	0.96 (0.70–1.30)	
rs13306553	TT	435 (80.3)	534 (81.7)	1.00	1.00	
	TC	105 (19.3)	116 (17.6)	1.06 (0.78–1.44)	0.97 (0.70–1.33)	
	CC	2 (0.4)	4 (0.6)	0.62 (0.11–3.60)	0.71 (0.11–4.55)	0.54
	TC + CC	107 (19.7)	120 (18.3)	1.05 (0.78–1.41)	0.96 (0.70–1.31)	
rs9651118	TT	230 (42.4)	273 (41.7)	1.00	1.00	
	TC	233 (43.0)	291 (44.5)	0.93 (0.72–1.20)	0.93 (0.71–1.21)	
	CC	79 (14.6)	90 (13.8)	1.05 (0.73–1.51)	1.00 (0.69–1.46)	0.66
	TC + CC	312 (57.6)	381 (58.3)	0.96 (0.75–1.21)	0.95 (0.74–1.21)	
rs1801133	CC	160 (29.5)	242 (37.0)	1.00	1.00	
	CT	270 (49.8)	308 (47.1)	1.34 (1.03–1.76)	1.31 (0.99–1.73)	
	TT	112 (20.7)	104 (15.9)	1.64 (1.16–2.31)	1.82 (1.27–2.61)	0.04
	CT + TT	382 (70.5)	412 (63.0)	1.42 (1.10–1.83)	1.44 (1.10–1.87)	
rs2274976	GG	433 (79.9)	542 (82.9)	1.00	1.00	
	GA	104 (19.2)	107 (16.4)	1.17 (0.86–1.60)	1.10 (0.80–1.52)	
	AA	5 (0.9)	5 (0.8)	1.34 (0.37–4.82)	1.50 (0.39–5.69)	0.21
	GA + AA	109 (20.1)	112 (17.1)	1.18 (0.87–1.60)	1.12 (0.81–1.53)	
rs4846048	AA	424 (78.5)	538 (82.3)	1.00	1.00	
	AG	108 (20.0)	111 (17.0)	1.23 (0.90–1.66)	1.19 (0.87–1.64)	
	GG	8 (1.5)	5 (0.8)	2.60 (0.81–8.38)	2.82 (0.83–9.54)	0.12
	AG + GG	116 (21.5)	116 (17.7)	1.28 (0.95–1.72)	1.25 (0.92–1.70)	
rs1801131	AA	333 (61.4)	448 (68.5)	1.00	1.00	
	AC	174 (32.1)	182 (27.8)	1.27 (0.97–1.64)	1.19 (0.91–1.57)	
	CC	35 (6.5)	24 (3.7)	1.97 (1.13–3.45)	1.99 (1.12–3.56)	0.03
	AC + CC	209 (38.6)	206 (31.5)	1.35 (1.05–1.73)	1.29 (0.99–1.66)	
rs17037396	CC	418 (77.1)	500 (76.3)	1.00	1.00	
	CT	112 (20.7)	142 (21.7)	0.95 (0.71–1.27)	0.96 (0.71–1.30)	
	TT	12 (2.2)	13 (2.0)	1.22 (0.54–2.79)	1.13 (0.49–2.62)	0.71
	CT + TT	124 (22.9)	155 (23.7)	0.97 (0.73–1.29)	0.97 (0.73–1.31)	

† Adjusted for age;

‡ Adjusted for age, cigarette smoking, diabetes and hypertension.

**Table 3.** Association of haplotypes in the *MTHFR* gene with risk of ischemic stroke.

Haplotype *	Cases, %	Controls, %	OR (95% CI) †	OR (95% CI) ‡
CTTTGA	38.7	38.7	1.00	1.00
CTCCGA	33.6	35.7	0.96 (0.79–1.17)	0.92 (0.76–1.12)
ATTCGC	6.8	8.7	0.79 (0.56–1.12)	0.76 (0.54–1.08)
ACTCAC	7.5	7.7	0.94 (0.67–1.33)	0.88 (0.62–1.24)
CTTCGA	3.2	6.6	0.55 (0.36–0.84)	0.53 (0.35–0.82)

* In the order rs12121543, rs13306553, rs9651118, rs1801133, rs2274976, rs1801131;

† Adjusted for age;

‡ Adjusted for age, cigarette smoking, diabetes and hypertension.

**Table 4.** Association between MTHFR polymorphisms and serum tHcy levels in healthy control.

SNP	M/m	tHcy (mmol/L)			*p*
			
		MM	Mm	mm	
rs12121543	C/A	12.4 (10.1–14.6)	12.1 (9.7–13.7)	10.8 (8.5–13.4)	0.26
rs13306561	T/C	12.1 (10.0–14.4)	12.3 (10.2–14.1)	9.1 (8.1–11.8)	0.45
rs13306553	T/C	12.1 (10.0–14.4)	12.4 (10.1–14.2)	9.1 (8.0–11.8)	0.45
rs9651118	T/C	12.5 (9.8–14.9)	12.0 (10.0–13.8)	11.5 (9.5–14.1)	0.02
rs1801133	C/T	10.9 (9.2–12.8)	12.8 (10.9–14.6)	13.1 (10.0–16.8)	<0.0001
rs2274976	G/A	12.1 (10.0–14.4)	12.3 (10.0–13.9)	9.8 (8.4–13.7)	0.57
rs4846048	A/G	12.3 (10.1–14.4)	11.6 (9.4–13.6)	11.1 (10.3–11.6)	0.36
rs1801131	A/C	12.4 (10.0–14.6)	12.2 (10.0–13.8)	10.4 (8.6–13.4)	0.36
rs17037396	C/T	12.2 (10.0–14.4)	11.9 (9.9–14.4)	11.7 (11.4–12.9)	0.94

M indicates major alleles; m indicates minor alleles.

## References

[1] Johnston S.C., Mendis S., Mathers C.D. (2009). Global variation in stroke burden and mortality: Estimates from monitoring, surveillance, and modelling. Lancet Neurol.

[2] Feigin V.L., Lawes C.M., Bennett D.A., Barker-Collo S.L., Parag V. (2009). Worldwide stroke incidence and early case fatality reported in 56 population-based studies: A systematic review. Lancet Neurol.

[3] Chen Z., Chen Z. (2008). The mortality and death cause of national sample areas. The Third National Survey on the Cause of Death.

[4] Zhang L.F., Yang J., Hong Z., Yuan G.G., Zhou B.F., Zhao L.C., Huang Y.N., Chen J., Wu Y.F. (2003). Proportion of different subtypes of stroke in China. Stroke.

[5] Yong H., Foody J., Linong J., Dong Z., Wang Y., Ma L., Meng H.J., Shiff S., Dayi H. (2013). A systematic literature review of risk factors for stroke in China. Cardiol. Rev.

[6] Shi H., Sheng B., Zhang F., Wu C., Zhang R., Zhu J., Xu K., Kuang Y., Jameson S.C., Lin Z. (2013). Kruppel-like factor 2 protects against ischemic stroke by regulating endothelial blood brain barrier function. Am. J. Physiol. Heart Circ. Physiol.

[7] Perry I.J., Refsum H., Morris R.W., Ebrahim S.B., Ueland P.M., Shaper A.G. (1995). Prospective study of serum total homocysteine concentration and risk of stroke in middle-aged British men. Lancet.

[8] Collaboration H.S. (2002). Homocysteine and risk of ischemic heart disease and stroke: A meta-analysis. JAMA.

[9] Low H.Q., Chen C.P., Kasiman K., Thalamuthu A., Ng S.S., Foo J.N., Chang H.M., Wong M.C., Tai E.S., Liu J. (2011). A comprehensive association analysis of homocysteine metabolic pathway genes in Singaporean Chinese with ischemic stroke. PLoS One.

[10] Frosst P., Blom H.J., Milos R., Goyette P., Sheppard C.A., Boers G.J., den Heijer M., Kluijtmans L.A., van den Heuvel L.P., Rozen R. (1995). A candidate genetic risk factor for vascular disease: A common mutation in methylenetetrahydrofolate reductase. Nat. Genet.

[11] Shahzad K., Hai A., Ahmed A., Kizilbash N., Alruwaili J. (2013). A structured-based model for the decreased activity of Ala222Val and Glu429Ala methylenetetrahydrofolate reductase (MTHFR) mutants. Bioinformation.

[12] Larsson S.C., Giovannucci E., Wolk A. (2006). Folate intake, MTHFR polymorphisms, and risk of esophageal, gastric, and pancreatic cancer: A meta-analysis. Gastroenterology.

[13] Yang S., Zhang J., Feng C., Huang G. (2012). MTHFR 677T variant contributes to diabetic nephropathy risk in Caucasian individuals with type 2 diabetes: A meta-analysis. Metabolism.

[14] Marosi K., Agota A., Végh V., Joó J.G., Langmár Z., Kriszbacher I., Nagy Z.B. (2012). The role of homocysteine and methylenetetrahydrofolate reductase, methionine synthase, methionine synthase reductase polymorphisms in the development of cardiovascular diseases and hypertension. Orvosi Hetil.

[15] Ye H., Yan J.T., Shao J.M., Zhang F., Hong M.L., Wang D.W. (2004). A case-control study on the relationship between stroke and serum homocysteine level and the mutation of *MTHFR* gene. Zhonghua Liu Xing Bing Xue Za Zhi.

[16] Shi C., Kang X., Wang Y., Zhou Y. (2008). The coagulation factor V Leiden, MTHFR C677T variant and eNOS 4ab polymorphism in young Chinese population with ischemic stroke. Clin. Chim. Acta.

[17] Li C.M., Zhang C., Lu X.L., Feng H.Y., Su Q.X., Zeng Y., Zhang H.L., Qiu S.L. (2006). Relationship between methylenetrahydrofolate reductase gene and ischemic stroke. Zhongguo Wei Zhong Bing Ji Jiu Yi Xue.

[18] Gao X., Yang H., Teng Z. (2006). Association studies of genetic polymorphism, environmental factors and their interaction in ischemic stroke. Neurosci. Lett.

[19] Wu J.M., Wang T.G., Li Y.Q., Song X.W., Liu Y.Y., Yun H.R., Zhong Z.Y., Zhou T.H. (2004). Genetic mutations of homocysteine metabolism related enzymes in patients with ischemic stroke. Yi Chuan.

[20] Jacques P.F., Bostom A.G., Williams R.R., Ellison R.C., Eckfeldt J.H., Rosenberg I.H., Selhub J., Rozen R. (1996). Relation between folate status, a common mutation in methylenetetrahydrofolate reductase, and plasma homocysteine concentrations. Circulation.

[21] Zhao X., Jiang H. (2013). Quantitative assessment of the association between MTHFR C677T polymorphism and hemorrhagic stroke risk. Mol. Biol. Rep.

[22] Hamzi K., Tazzite A., Nadifi S. (2011). Large-scale meta-analysis of genetic studies in ischemic stroke: Five genes involving 152,797 individuals. Indian J. Hum. Genet.

[23] McColgan P., Sharma P. (2008). The genetics of carotid dissection: Meta-analysis of a MTHFR/C677T common molecular variant. Cerebrovasc. Dis.

[24] Somarajan B.I., Kalita J., Mittal B., Misra U.K. (2011). Evaluation of MTHFR C677T polymorphism in ischemic and hemorrhagic stroke patients. A case-control study in a Northern Indian population. J. Neurol. Sci.

[25] Kim N.K., Choi B.O. (2003). MTHFR A1298C gene polymorphism: Independent risk factor for ischemic stroke?. Korean Neurol. Assoc. J.

[26] Han I.B., Kim O.J., Ahn J.Y., Oh D., Hong S.P., Huh R., Chung S.S., Kim N.K. (2010). Association of methylenetetrahydrofolate reductase (MTHFR 677C>T and 1298A>C) polymorphisms and haplotypes with silent brain infarction and homocysteine levels in a Korean population. Yonsei Med. J.

[27] Arsene D., Găină G., Bălescu C., Ardeleanu C. (2011). C677T and A1298C methylenetetrahydropholate reductase (MTHFR) polymorphisms as factors involved in ischemic stroke. Rom. J. Morphol. Embryol.

[28] Kumar J., Das S.K., Sharma P., Karthikeyan G., Ramakrishnan L., Sengupta S. (2005). Homocysteine levels are associated with MTHFR A1298C polymorphism in Indian population. J. Hum. Genet.

[29] Narayanan S., McConnell J., Little J., Sharp L., Piyathilake C.J., Powers H., Basten G., Duthie S.J. (2004). Associations between two common variants C677T and A1298C in the methylenetetrahydrofolate reductase gene and measures of folate metabolism and DNA stability (strand breaks, misincorporated uracil, and DNA methylation status) in human lymphocytes *in vivo*. Cancer Epidemiol. Biomark. Prev..

[30] Swartz M.D., Peterson C.B., Lupo P.J., Wu X., Forman M.R., Spitz M.R., Hernandez L.M., Vannucci M., Shete S. (2013). Investigating multiple candidate genes and nutrients in the folate metabolism pathway to detect genetic and nutritional risk factors for lung cancer. PLoS One.

[31] Yoshimi A., Aleksic B., Kawamura Y., Takahashi N., Yamada S., Usui H., Saito S., Ito Y., Iwata N., Inada T. (2010). Gene-wide association study between the methylenetetrahydrofolate reductase gene (MTHFR) and schizophrenia in the Japanese population, with an updated meta-analysis on currently available data. Schizophr. Res.

[32] Marti F., Vollenweider P., Marques-Vidal P.M., Mooser V., Waeber G., Paccaud F., Bochud M. (2011). Hyperhomocysteinemia is independently associated with albuminuria in the population-based CoLaus study. BMC Public Health.

[33] Barrett J.C., Fry B., Maller J., Daly M.J. (2005). Haploview: Analysis and visualization of LD and haplotype maps. Bioinformatics.

[34] Lin D.Y., Zeng D., Millikan R. (2005). Maximum likelihood estimation of haplotype effects and haplotype-environment interactions in association studies. Genet. Epidemiol.

